# From research to practice

**DOI:** 10.1186/1940-0640-8-S1-A36

**Published:** 2013-09-04

**Authors:** Craig Jones, Sarah Jones, Paul Jordan, Carol Foster, John Bradley

**Affiliations:** 1 Public Health Wales, Cardiff, UK

## 

Alcohol misuse poses a serious public health challenge in Wales. The Public Health Wales alcohol team is working across Wales to reduce alcohol related harm, both acute and chronic, in the Welsh population. In order to achieve this aim, Pubic Health Wales has trained over 3000 people to deliver alcohol brief interventions. The team has diversified its target audience to encompass anyone who has regular contact with people who may have an alcohol problem. The ethos of the programme is that the reduction in the harm caused by alcohol is everybody's problem, not just primary care. Trusting the evidence base of an NNT of 1 in 8, over 200 drinkers per month are shifting their behavior from hazardous and harmful drinking back towards being sensible drinkers. It has emerged that certain professionals (midwives, health visitors, youth workers, custody officers) are well placed to deliver brief interventions and that a 'blanket' approach to training results in more brief interventions being delivered than ad hoc training.

